# Concomitant angiosarcoma and lymphoproliferative disorder in solid organ transplant recipients

**DOI:** 10.1186/2045-3329-4-15

**Published:** 2014-10-31

**Authors:** Lea N Baer, David G Savage, Hanina H Hibshoosh, Kevin Kalinsky

**Affiliations:** Division of Hematology and Oncology, Stony Brook Medicine, New York, NY USA; Division of Hematology and Oncology, New York- Presbyterian, New York, NY USA; Department of Pathology, New York-Presbyterian, New York, NY USA

**Keywords:** Angiosarcoma, Posttransplant lymphoproliferative disorder, Solid organ transplant

## Abstract

An increased risk of posttransplant malignancy has been consistently reported following various solid organ transplants. The malignancies most commonly encountered are non-melanoma skin cancers, carcinomas of lung or breast and posttransplant lymphoproliferative disorders. Angiosarcoma, an uncommon vascular mesenchymal neoplasm, is rare in the posttransplant setting. This report describes two patients who developed high-grade angiosarcoma following a solid organ transplant. Notably, in both patients, the diagnosis of angiosarcoma was preceded by diagnosis of a lymphoproliferative disorder with monoclonal immunoglobulin heavy chain rearrangement.

## Background

An increased risk of developing malignancy has been observed following solid organ transplantation, with rates and malignancy type closely related to the transplanted organ. The most common neoplasms encountered are non-melanoma skin cancers (NMSC). Significant increases in incidence of other common malignancies such as lung, breast, and renal cell carcinomas are consistently reported as well [1]. While posttransplant lymphoproliferative disorder (PTLD) is one of the more common malignancies developing in solid organ transplant recipient, angiosarcoma is rare in this setting. We describe two patients diagnosed with concomitant PTLD and angiosarcoma following solid organ transplant.

### Case I

**Figure 1:**
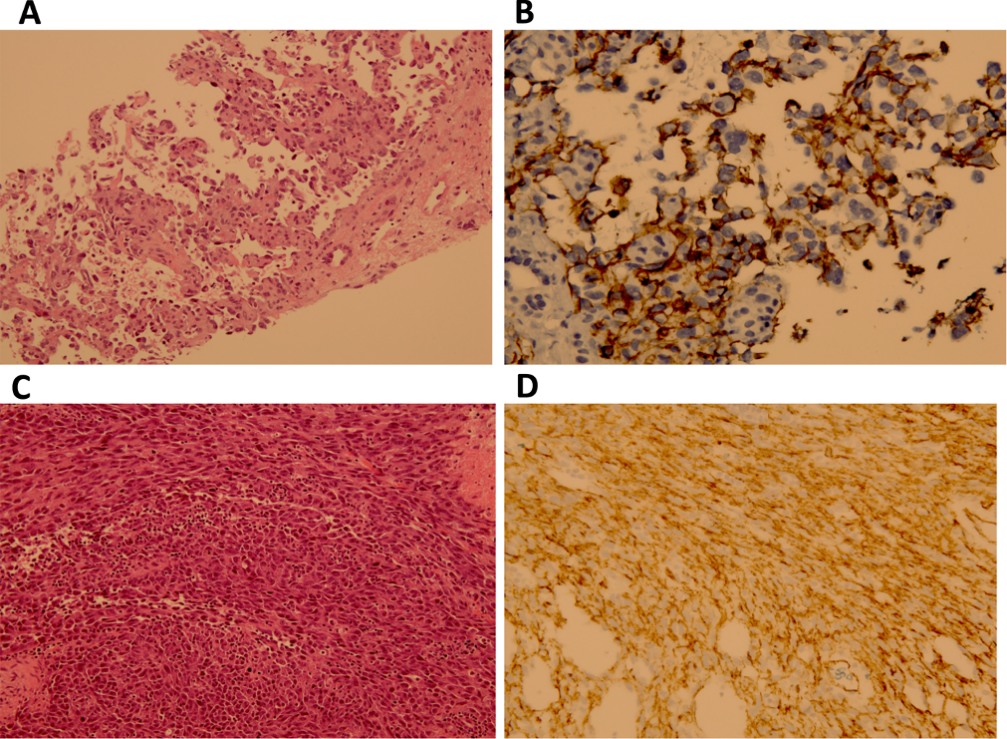
**Histopathology of patients with angiosarcoma in the setting of solid organ transplant. A)** CM angiosarcoma 200x: Needle core biopsy of the liver showing inter-anastomosing channels with papillary formation lined by atypical endothelial cells typical of angiosarcoma (200X). **B)** CM angiosarcoma 400X CD31: Immunohistochemical stain against CD31(endothelial marker) demonstrates with brown staining the neoplastic endothelial cells of this angiosarcoma (400X). **C)** KM angiosarcoma 200X: A high grade angiosarcoma composed of cellular spindle malignancy growing in fascicles with multiple mitotic figures with scattered red blood cells but without evidence of vascular formation (200X). **D)** KM CD31 400X: Immunohistochemical stain against CD31 (endothelial marker) demonstrates with brown staining the neoplastic endothelial cells of this high-grade angiosarcoma (400X).

62-year-old man with alcoholic cirrhosis initially treated with chemoembolization for hepatocellular carcinoma. He underwent orthotopic liver transplant in January 2006. Following transplant, he was maintained on the immunosuppressant tacrolimus. A routine surveillance MRI performed two years later (April 2008) revealed a 3.5 cm left adrenal mass. Systemic imaging, including PET/CT, reported an adrenal mass, splenomegaly (18 cm) and FDG-avid mesenteric and para-aortic lymphadenopathy. Biopsy of the left adrenal mass showed a post-transplant lymphoproliferative disorder (PTLD), consistent with an Epstein-Barr virus (EBV) negative, monomorphic, diffuse large B-cell lymphoma. Spinal fluid and bone marrow were both unremarkable. EBV DNA was undetectable by PCR. The tacrolimus dose was decreased by half, and the patient was started on CHOP chemotherapy (cyclophosphamide, doxorubicin, vincristine and prednisone) with rituximab. Interim evaluation with a PET/CT showed disease progression and treatment was switched to the ICE regimen (ifosfamide, carboplatin and etoposide) with rituximab. Autologous stem cells were harvested and stored after his ICE chemotherapy. Upon completion of chemotherapy, PET/CT demonstrated disease remission and bone marrow biopsy remained negative for lymphoma. Five months later, the patient developed a firm swelling of his upper lip. A biopsy revealed monophasic diffuse large-B-cell lymphoma with clonal heavy chain rearrangement. A PET/CT showed FDG avidity in the upper lip and sub-mandibular lymph node. Due to concern for significant local morbidity, he was treated with dexamethasone and a palliative course of radiation. In view of limited improvement, the radiation was discontinued after only four fractions. He then received 2 additional cycles of ICE with rituximab. In April 2009, the patient was admitted for autologous stem cell transplant, following etoposide, carboplatin and thiotepa conditioning. He tolerated the autologous transplant well, but a subsequent PET/CT showed continued FDG avidity in the upper lip. Local radiation therapy was resumed. Following stem cell transplant, he was maintained on rituximab every three months, the last of which was given in November 2010.In February 2011, he presented with dyspnea on minimal exertion, abdominal dissension, malignant ascites, and disseminated intravascular coagulation. Imaging revealed a cirrhotic liver, portal hypertension, and a new 23 cm hepatic mass with hemoperitoneum. Biopsy of the liver mass revealed highly atypical cells with hyperchromasia and markedly pleomorphic nuclei with prominent nucleoli and markedly increased mitotic activity including atypical mitotic figures (Figure 1A). Immunohistochemical assessment revealed tumor cells to be positive for CD31 (Figure 1B) and focally positive for CD34, suggestive of high-grade angiosarcoma. The cells were negative for hepatocellular specific antigen and Human Herpes virus 8 (HHV-8). EBV DNA was undetectable by PCR. His hospital stay was further complicated by respiratory failure and renal failure requiring initiation of dialysis. The patient passed away prior to initiation of systemic therapy.

### Case II

A 25-year-old female with a prolonged history of gastroparesis secondary to intestinal neuronal dysplasia. After presenting with intestinal pseudo-obstruction in May 2006, she underwent multiple surgical interventions, including a partial colectomy, lysis of adhesions, resection of ilieo-colic anastomosis, and repeat ilieo-colic anastomosis. In March 2009, she presented with intestinal dysmotility and liver failure, ultimately undergoing multi-visceral transplantation (en bloc transplant of stomach, pancreas, liver, small bowel, and colon). Following the transplant, she was maintained on rapamycin for immunosuppression. EBV DNA copies were not detected prior to transplant but quickly rose to 3,500 copies following transplant and upon initiation of immune suppression. Her viral level became undetectable on anti-viral therapy.In September 2012, she had a colostomy surgery due to abdominal distention and colonic dysmotility. At the same time, an enlarged left cervical node was noted, and a biopsy was performed. Pathology review of the specimen showed a focal increase in large B cell population, found to be distributed in loose nodules concerning for PTLD. Flow cytometry was attempted but failed due to the low cellularity of the specimen. However, a clonal immunoglobulin heavy chain gene rearrangement was detected by PCR, further supporting the possibility of PTLD. In November 2012, the patient noted rapidly increasing abdominal girth. CT imaging demonstrated ascites and a 4.8cm ill-defined soft tissue density in the left suprarenal area. In January 2013, she underwent resection of the left adrenal mass, a mass around the splenic bed, and of an abdominal deposit. Pathology of these areas showed high-grade angiosarcoma with positive surgical margins (Figure 1C). Immunohistochemical stains were positive for CD34, CD31 (Figure 1D), CAN5.2, factor VIII, c-KIT and inhibin. HHV-8 immunostaining of the tumor was negative. Post-surgical CT scan on 2/2013 showed no evidence of residual disease. The hospital course was further complicated by rejection, treated with anti-thymocyte globulins (ATG) and steroids. Her condition continued to worsen with dyspnea and new inferior vena caval and peroneal venous thrombosis. The patient and family opted for palliative care, and she passed away prior to systemic therapy.

## Conclusions

An increased risk of developing malignancy has been consistently observed following solid organ transplantation, with rates and malignancy type varying on the transplanted organ [1]. Non-melanoma skin cancers (NMSCs) are the most common malignancy in transplant recipients and affect up to 10-45% of transplant recipients, depending on race and ultraviolet light exposure [1]. Squamous cell carcinoma is the most common NMSC. Recipients of renal transplants, the most common solid organ to be transplanted, are the best studied to date in the context of post-transplant malignancies. In renal transplant recipients, the incidences of the most common cancers (colon, lung, prostate and breast) are roughly two-fold higher in the first 3 years after kidney transplantation, when compared to the general population [2]. Kaposi’s sarcoma and non-Hodgkin’s lymphoma are more than 20-fold higher than in the general population. When compared to patients on transplant waiting lists, several malignancies rates are more common: NMSCs (2.6-fold), melanoma (2.2-fold), Kaposi’s sarcoma (9.0-fold) and non-Hodgkin’s lymphoma (3.3-fold). Angiosarcoma is not reported as a unique entity in these studies and is included in the non-skin malignancies not otherwise specified (NOS) category which accounts for about 3% of the malignancies developing in the first 3 years following kidney transplant.

The relative rate of each post-transplant malignancy varies depending on the transplanted solid organ. In 2011, the U.S. Organ Procurement and Transplantation Network and the Scientific Registry of Transplant Recipients reported a 2% incidence of PTLD at 5 years post renal-transplant in EBV-negative adult recipients [3]. Following liver transplant, the rate of PTLD is 10.2% at 5 years, with slightly higher rates in EBV-negative recipients. In contrast, PTLD following heart transplant is relatively infrequent in adult recipients and closely linked to EBV status. Risk of malignancy of any kind is especially high following lung transplant and has been reported to be as high as 15.4% at 5 years post-transplant. These observed differences in risk may be due to variations in length and type of immunosuppression given to recipients of the various solid organs.

Immunosuppression is often cited as the etiology of the increased risk of malignancy, possibly by conferring susceptibility to viruses implicated in oncogenesis such as EBV, herpes simplex, herpes zoster, HHV8 and polyoma virus [4]. Other potential mechanisms include direct oncogenesis by the immunosuppressive agents themselves; for example, the chromosome breaks and nuclear abnormalities observed with azathioprine use [5]. Hepatic angiosarcoma developing following an extensive cyclophosphamide exposure was reported in a patient treated for polyarteritis nodosa [6]. In kidney transplant recipients, immunosuppression with cyclosporine did not confer added risk when compared with azathioprine/steroid treatment, whereas treatment with tacrolimus has been shown to increase the risk approximately two-fold. Induction or anti-rejection therapy with OKT3 or ATG increases the risk of lymphoma during the first post-transplant year [7].

Sarcomas are uncommon mesenchymal malignancies and comprise less than 1% of malignant neoplasms in adults. 80% of them arise in soft tissues [8, 9]. Angiosarcoma is an uncommon type of soft tissue sarcoma, which may rise in a variety of tissues or organs. A well-documented risk factor for developing angiosarcoma is therapeutic radiation, such as the risk observed 8–10 years after breast radiotherapy [10]. Angiosarcoma has a propensity for early metastases and often presents with an aggressive biologic behavior. In a series of 67 patients with angiosarcomas, the actuarial 5 year disease-free-survival is 35% and the 5 year overall survival is 24% [8]. In another series of 82 patients with angiosarcoma, the five-year disease-specific survival is found to be 60%, with negative prognostic variables including higher tumor grade and tumors arising in areas of lymphedema or prior irradiation [9]. Similar to the majority of soft tissue sarcomas, the mainstay of therapy is complete surgical resection of the tumor, if operable. Adjuvant therapies such as post-operative radiation to the tumor bed with wide margins are offered to patients with tumors larger than 5cm or to those with positive surgical margins [11]. The role of adjuvant chemotherapy remains controversial in soft tissue sarcoma due to lack of consistent survival benefit in studies published to date [12]. Newer studies evaluating paclitaxel and bevacizumab in patients with unresectable or metastatic angiosarcoma suggest a possible role for these to treat angiosarcoma [13, 14].

Post-transplant angiosarcomas are rare and, to date, have been reported in less than 20 solid organ transplant recipients. In a single-institution prospective database encompassing 1,570 liver transplantations in 1,421 patients from 1985 to 1999, 125 patients (8.8%) developed de novo tumors, one of them being angiosarcoma [15]. The majority of reported patients with post-transplant angiosarcoma have been renal transplant recipients with tumor frequently developing at the site of a previously placed arterio-venous fistula [16]. An association has been suggested between angiosarcoma and oncoviruses, such as HHV-8, following detection of a viral sequence in the DNA of a primary angiosarcoma [17]. This association remains controversial, as others have failed to detect similar sequences in either primary [18] or in post-transplant angiosarcoma [19]. In a series of 3 patients with PTLD reported by Webster et al., 2 of the patients have PTLD as well as angiosarcoma [16]. In this series, 1 patient has been identified with an EBV-positive tumor and a second with an elevated EBV titer at the time of diagnosis of disseminated angiosarcoma. The 2 patients in our paper both had angiosarcomas diagnosed following a prior diagnosis of PTLD, and 1 had a positive EBV titer detected at the time of angiosarcoma diagnosis. Both angiosarcomas were found to be negative for HHV-8 immunostaining.

In summary, the few reported patients with angiosarcoma and PTLD vary in type of transplanted organ, immunosuppression therapy and in seropositivity for a possible oncogenic virus. While the possible association of the rare post-transplant angiosarcomas with PTLD may suggest the presence of underlying host variables predisposing the recipients to develop post-transplant malignancies, these are yet to be defined. Assessment of genetic abnormalities in these tumors in the future may inform the possible susceptibility conferring host variables.

### Consent

Written informed consent was obtained from the patient for publication of this Case Report and any accompanying images. A copy of the written consent is available for review by the Editor-in-Chief of this journal.

## Abbreviations

PTLD: Posttransplant lymphoproliferative disorder
EBV: Epstein-Barr virus
ATG: Anti-thymocyte globulins
STS: Soft tissue sarcoma
MRI: Magnetic resonance imaging
CT: Computed tomography
PET: Positron emission tomography
FDG: Fludeoxyglucose
PCR: Polymerase chain reaction
NMSC: Non-melanoma skin cancers.
